# Emergent Management of Traumatic Posterior Sternoclavicular Joint Dislocation: A Case Report and Literature Review

**DOI:** 10.7759/cureus.18996

**Published:** 2021-10-23

**Authors:** Xi Chen, Dylan Shafer, Arianna S Neeki, Fanglong Dong, James Matiko, Michael M Neeki

**Affiliations:** 1 Emergency Medicine, California University of Science and Medicine, Colton, USA; 2 College of Osteopathic Medicine of the Pacific, Western University of Health Sciences, Pomona, USA; 3 Emergency Medicine, Arrowhead Regional Medical Center, Colton, USA; 4 Graduate College of Biomedical Sciences, Western University of Health Sciences, Pomona, USA

**Keywords:** computed tomography, case report, closed reduction, sternoclavicular joint, traumatic sternoclavicular joint dislocation

## Abstract

Sternoclavicular joint (SCJ) dislocation is a rare but serious orthopedic injury. Posterior dislocations are more concerning due to the SCJ’s proximity to visceral structures such as the trachea, esophagus, subclavian vessels, and brachial plexus. Due to the potential long-term sequelae of missed diagnosis, clinical suspicion should be high when a patient presents with a compression-type injury to the shoulder girdle and pain or deformity to the SCJ.

Here we present a case of a 15-year-old soccer player who presented to the emergency department (ED) after a fall onto his right shoulder with additional compound injuries. A posterior SCJ dislocation diagnosis was suspected and confirmed after a computed tomography scan. A successful closed reduction was done in the ED after consultation with cardiothoracic and orthopedic surgery.

This case adds to the body of literature describing diagnosis and management of posterior SCJ dislocations.

## Introduction

Sternoclavicular joint (SCJ) dislocation is a relatively uncommon injury despite being one of the least stable joints in the body due to the relatively small articular contact between the sternum and the clavicle. SCJ dislocations account for 3% of all dislocations associated with the shoulder and can be classified into anterior and posterior dislocations [1]. Posterior dislocations are less frequent, representing 3%-5% of SCJ dislocations [2].

The SCJ’s stability is achieved primarily with the costoclavicular and interclavicular ligaments in addition to the capsule. Because only 50% of the medial clavicular surface articulates with the sternum, most of the stability is derived from the surrounding ligamentous structures [1]. Cadaveric studies have shown the posterior aspect of the capsule is the strongest ligamentous stabilizer, particularly in the anterior-posterior direction [3]. Patients with SCJ dislocations typically present after an indirect force with lateral compression such as falling onto the shoulder or lateral impact in motor vehicle accidents [2]. The most common presentations are pain over the SCJ and inability to abduct the ipsilateral shoulder due to SCJ's articulation between axial and appendicular skeleton [1].

In addition to pain and decreased range of motion, posterior SCJ dislocations specifically may interfere with visceral structures such as the trachea, esophagus, subclavian vessels, and supraclavicular brachial plexus [1,3]. Thus, patients may also present with stridor, paresthesias, hoarseness, dyspnea, dysphagia, and cardiac conduction abnormalities, which can be life-threatening [1]. Due to the proximity to essential structures, posterior SCJ dislocations are typically treated with either closed or open reduction [4].

Due to the rare nature of posterior SCJ dislocations, emergency medicine physicians may have little experience managing this pathology. We present a novel case of a 15-year-old male who sustained a posterior SCJ dislocation with subsequent closed reduction done in the emergency department (ED). This study was approved by the Institutional Review Board at Arrowhead Regional Medical Center, Colton, California, with the IRB approval number 21-30.

## Case presentation

A 15-year-old male soccer player was transported by the Emergency Medical Services (EMS) from an outside facility to the ED of a regional trauma center complaining of right shoulder and clavicular pain after sustaining a lateral fall onto artificial turf as a goalkeeper. While on the ground, another player fell onto the patient, causing compounded bilateral compressing forces to the upper thoracic region of the patient. The patient heard a painful pop accompanied by severe pain in his right upper chest and shoulder region. He was unable to move his shoulder and was reportedly holding his arm in an adducted and internally rotated position. EMS placed a sling on the patient prior to transport to the hospital.

**Figure 1:**
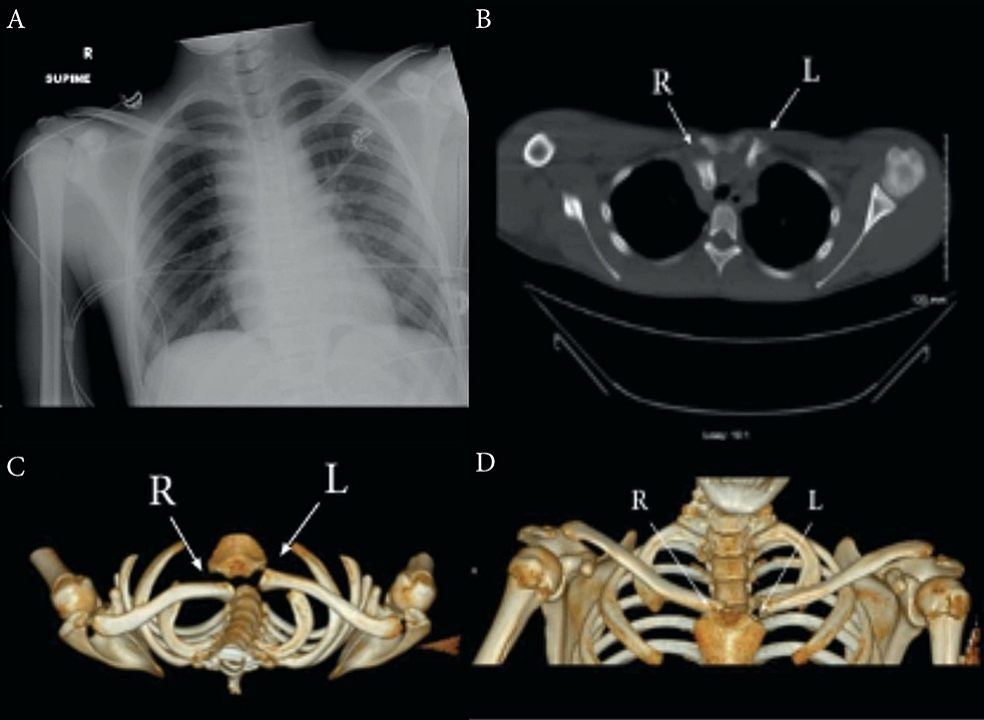
A) Anterior-posterior X-ray of the chest on arrival. B) Axial CT view demonstrating posterior dislocation of the right sternoclavicular joint. C) Axial three-dimensional (3D) CT reconstruction. D) Coronal 3D CT reconstruction. CT: computed tomography, R: right, L: left

On the initial survey, the patient had blood pressure of 138/89 millimeters of mercury (mmHg), Glasgow Coma Scale of 15 (Eye 4, Verbal 5, Motor 6), and pulse oximetry of 100% on room air. There was no trauma to the head, tenderness over the spine or step-off noted. The patient had no obvious airway compromise. Upper extremity pulses were 2+ bilaterally. However, a visual inward deformity at the right SCJ was noted. Standard radiographic images were obtained and demonstrated a posterior misalignment of the clavicle with no apparent tracheal deviation. Suspecting SCJ dislocation, computed tomography (CT) scan without contrast was performed and confirmed the diagnosis of right SCJ dislocation (Figure 1). No further mediastinal injuries were noted on the CT scan.

**Figure 2:**
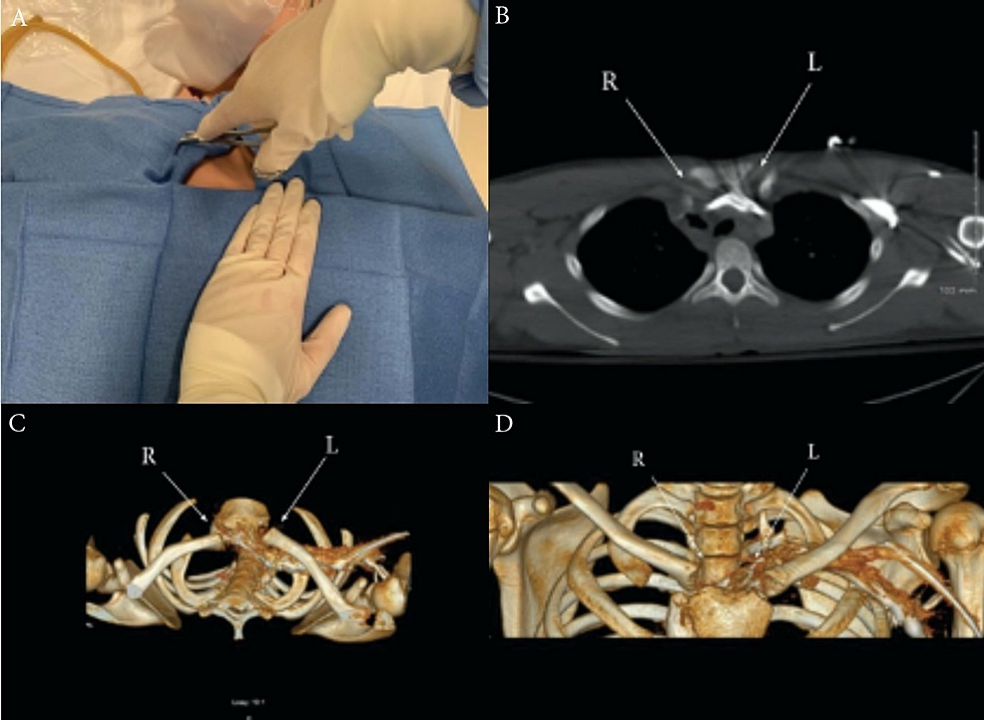
A) Reduction technique using percutaneous towel clamp forceps engaging the medial aspect of the dislocated clavicle while the other hand stabilizing the sternum. B) Axial CT showing anatomical reduction of posterior dislocation of the right sternoclavicular joint. C) Post-reduction axial CT reconstruction. D) Post-reduction coronal CT three-dimensional (3D) reconstruction. CT: computed tomography, R: right, L: left

In addition, the underlying major vessels appeared to be undisturbed by the posterior dislocation of the clavicle. In congruence with cardiovascular and orthopedic surgery recommendations, a closed reduction was performed using procedural sedation by administering midazolam 2 milligram (mg) intravenous (IV), fentanyl 75 microgram (mcg) IV and propofol 30 mg IV in three repeated increments for each medication. Initially, the ED team attempted a reduction using longitudinal traction with 90-degree arm abduction and extension while the patient was in the supine position. A log roll was placed under the patient's scapula for improved anterior distraction. Due to failure to reduce the dislocation using the aforementioned technique, the medial aspect of the clavicle was prepped and draped, and sterile towel clamps were inserted percutaneously. With steady anterior traction using the towel clamp and longitudinal traction of the arm, realignment of the clavicular head was achieved and confirmed with a post-reduction CT scan (Figure 2). The patient was subsequently discharged after a three-hour observation period in the ED and recommended to follow up with outpatient primary care and orthopedic services based on their private insurance plan.

## Discussion

SCJ dislocations are typically delayed diagnoses due to their rare, subtle presentation, as well as their nonspecific findings on standard radiography. SCJ dislocations may present with additional symptoms other than pain over the joint and decreased range of motion of the ipsilateral shoulder due to compressed mediastinum structures [5,6]. First-line diagnostic imaging includes standard radiographs. However, sternoclavicular visualization is difficult due to overlapping shadows of the rib, sternum, and clavicle at the joint [6,7]. Orthopedic consultants usually suggest additional views such as the serendipity view, where the x-ray beam is positioned at a 40-degree cephalic tilt for better visualization of the SCJ [1].

The choice of diagnostic imaging is vital in managing posterior SCJ dislocations. Life-threatening complications may result from prolonged compression of the mediastinum structures if the injury is left undiagnosed and untreated. The importance of an accurate diagnosis is highlighted in a case report by Marcus et al. [6]. A 19-year-old patient with a posterior SCJ dislocation was diagnosed as having a "pectoral strain" after negative findings on standard radiographs and was discharged home without further CT scan or consultation with orthopedic services. Later, the patient presented with middle cerebral artery distribution ischemia, which resulted from a pseudoaneurysm in the innominate artery due to compression from a posteriorly dislocated SCJ. This case report demonstrates that posterior SCJ dislocations are a potentially life-threatening injury and require a high index of suspicion. If a posterior SCJ dislocation is suspected, a CT scan is currently considered the gold standard for establishing the diagnosis [1]. We also followed the current guideline and the diagnosis was confirmed after the patient underwent the CT scan.

The treatment recommendation for posterior SCJ dislocations is conservative management by attempting closed reductions. A systematic review of 251 cases of SCJ dislocations reported that the closed reduction method achieved better functional outcomes than the open reduction method [8]. Although the posterior SCJ dislocation reductions in most case reports were performed by orthopedic surgeons, it is within the scope of practice of the emergency medicine physicians to perform this procedure if there is emergent neurovascular or airway compromise on presentation [9]. In our case, the procedure was performed by an experienced emergency medicine physician with prior encounters with similar cases. Anterior traction was applied to the medial clavicle using sterile towel clamps inserted percutaneously along with longitudinal traction of the ipsilateral arm. The reduction was performed under procedural sedation in the emergency department compared to general anesthesia used in the operating room which is typical in other reported cases [10].

The choice and location of reduction depend on the circumstances and stability of the patient. If the patient is unstable due to the involvement of mediastinum structures, a closed reduction can be attempted in the ED under procedural sedation either by orthopedic or emergency medicine physicians [9]. If the patient is stable and the orthopedic consultant is readily available or if a reduction attempt has failed in the ED, posterior SCJ dislocation reductions can be performed in the operating room with either procedural sedation or general anesthesia [8]. Some literature suggests that it is crucial to have cardiothoracic surgery on call for reductions in case of mediastinal complications during the procedure especially for posterior SCJ dislocation [11]. The majority (96% of the cases) of reduced posterior dislocations are stable and achieve good outcomes [8].

After reduction, the patient is usually placed in a shoulder sling or figure-of-eight brace with the recommendation of ice and anti-inflammatories to reduce swelling and manage pain [12]. Range of motion can be attempted after three to four weeks post-reduction [12]. Although the majority of the patients are treated conservatively and do well, some patients may experience recurrent instability, persistent pain and scapular dyskinesia due to instability or locked dislocation of the SCJ and may need to be treated operatively [11,13]. The operative management of SCJ dislocation can be achieved using gracilis tendon graft and figure-eight suture fixation [13]. Patients with successful closed reduction with recurrent dislocation can further be treated with locking plate osteosynthesis or open reduction and internal fixation using K-wires, tension band wires or hook plates to provide stability [14,15]. There is a limited number of reported open reduction cases in the literature. However, the majority of the suture fixation, tendon graft and open reduction and internal fixation had optimal results with low frequency of high-risk complications [8].

## Conclusions

This report described a unique case of a young teenager who experienced posterior SCJ dislocation, which may be a potentially life-threatening injury and require a high index of suspicion. This case adds to the body of literature on posterior SCJ dislocations and may help guide decision-making in terms of acute and follow-up management. Emergency physicians should have a high suspicion of this particular injury in selective patients with injury mechanisms consistent with reported posterior SCJ dislocations. In addition, the emergency reduction procedure should take place in consultant with sub-specialty support.
